# Optimization of Total Phenolic and Flavonoid Contents of Defatted Pitaya (*Hylocereus polyrhizus*) Seed Extract and Its Antioxidant Properties

**DOI:** 10.3390/molecules25040787

**Published:** 2020-02-12

**Authors:** Siti Atikah Zulkifli, Siti Salwa Abd Gani, Uswatun Hasanah Zaidan, Mohd Izuan Effendi Halmi

**Affiliations:** 1 Halal Products Research Institute, Universiti Putra Malaysia, Putra Infoport, 43400 Serdang, Selangor, Malaysia; sitiatikahzulkifli@gmail.com; 2 Department of Agriculture Technology, Faculty of Agriculture, Universiti Putra Malaysia, 43400 Serdang, Selangor, Malaysia; 3 Department of Biochemistry, Faculty of Biotechnology and Biomolecular Sciences, Universiti Putra Malaysia, 43400 Serdang, Selangor, Malaysia; uswatun@upm.edu.my; 4 Department of Land Management, Faculty of Agriculture, Universiti Putra Malaysia, 43400 Serdang, Selangor, Malaysia; m_izuaneffendi@upm.edu.my

**Keywords:** defatted pitaya seed, extraction, phenolic content, flavonoid content, antioxidant activity, response surface methodology

## Abstract

The present study was conducted to optimize extraction process for defatted pitaya seed extract (DPSE) adopting response surface methodology (RSM). A five-level central composite design was used to optimize total phenolic content (TPC), total flavonoid content (TFC), ferric reducing antioxidant power (FRAP), and 2,2′-azino-bis (3-ethylbenzothizoline-6-sulfonic acid (ABTS) activities. The independent variables included extraction time (30–60 min), extraction temperature (40–80 °C) and ethanol concentration (60%–80%). Results showed that the quadratic polynomial equations for all models were significant at (*p* < 0.05), with non-significant lack of fit at *p* > 0.05 and R^2^ of more than 0.90. The optimized extraction parameters were established as follows: extraction time of 45 min, extraction temperature of 70 °C and ethanol concentration of 80%. Under these conditions, the recovery of TPC, TFC, and antioxidant activity based on FRAP and ABTS were 128.58 ± 1.61 mg gallic acid equivalent (GAE)/g sample, 9.805 ± 0.69 mg quercetin equivalent (QE)/g sample, 1.23 ± 0.03 mM Fe2+/g sample, and 91.62% ± 0.15, respectively. Ultra-high-performance liquid chromatography-quadrupole time-of-flight mass spectrometry (UPLC-QTOF/MS) analysis identified seven chemical compounds with flavonoids constituting major composition of the DPSE.

## 1. Introduction

Pitaya, *Hylocereus polyrhizus,* or commonly known as dragon fruit in English or ‘Buah Naga’ in Malay, is oblong in shape with scaly structures on its outer peels. It belongs to the family, Cactacae from the genus *Hylocereous*. Pitaya originates from Central and Northern South America and presently has become a recent fruit crop of interest being cultivated in Malaysia, Thailand, Vietnam, Australia, Taiwan, and other regions around the world. Other than appealing appearance of the fruit, pitaya has recently gained much attraction due to its high dietary contents [1,2]. The flesh of the fruit is tasty and sugary with numerous tiny and grainy black seeds. Pitaya fruit seeds contain oil like most other fruit seeds such as seeds of grape [3], linseeds [4] and berry including blackberry, blueberry and red raspberry seeds) [5]. Seed oil from pitaya fruit has been successfully extracted and reported to be potentially suitable in food, health and cosmetic applications. However, biological activities of polyphenols extracted from defatted pitaya seed (DPS) as a byproduct after extraction, still remains unexplored.

Polyphenols or phenolic compounds are plants’ secondary metabolites produced to shield plants from ultraviolet rays or damaging insect pests or disease pathogens. Present in plant tissues, they play important roles in plants’ nutrients assimilation, protein synthesis, enzyme activities, photosynthesis, cell signaling, and protection against adverse environmental conditions [6]. Flavonoids are one of the important classes of natural compounds which encompass the largest group of plant polyphenols. The compounds are made up of fifteen carbon atoms having common basic structures of two aromatic rings bound together by three carbon atoms. This group of compounds is classified as low molecular weight compared to other groups of polyphenols that are widely distributed in plants. There are several classes of flavonoids, such as flavonols, flavones, flavanones, flavanols, isoflavones, and flavanonols. 

Flavones and flavonols are the most abundantly present in most plants [7]. A number of studies have reported medicinal features of this group of polyphenols such as anticancer [8], anti-inflammatory, antimicrobial and antioxidant activities [9]. The main mechanism for these activities comprises of the reduction of oxidative stress by scavenging free radicals [10]. It had been documented that the mechanism was either by transferring electron to complete the radicals compound or by breaking them down to make them safer and become more stable [11]. A number of extensive research works had been conducted to extract phenolic and flavonoid compounds from natural sources for their potentially high therapeutic properties.

Extraction process is the primary stage to source crude extract of bioactive compounds from plant materials. Various plant materials require distinct extraction conditions and procedures to yield optimum retrieval of phenolic compounds as every single plant has distinctive characteristics in terms of phenolic constituents [12]. Several factors have been proven to significantly affect extraction yield such as extraction methods, particle size, solvent types, solvent concentrations, solvent-to-solid ratios, extraction temperatures, extraction times, and pH levels [13,14,15]. One-variable-at-a-time approach is a traditional method of analyzing extraction optimization conditions by changing only one factor at a time while keeping others set at constant values. However, this approach is time-consuming and required a large number of experiments and materials. Moreover, interactive effect between the factors studied cannotbe determined. Thus, experimental design has been used to overcome these problems. 

Experimental design is a systematic approach to apply statistical methods in experimental processes for various area of academic research and industry. The most common statistical techniques used to optimize the process is response surface methodology (RSM). RSM is an integrated statistical and mathematical technique to determine the relationship between the independent variables and dependent variable based on experimental design. That is, RSM has become an important tool that is employed for modeling by identifying the influence of several factors on the process. This method was first developed by Box and Wilson [16] to optimize the chemical process and now has been extensively adopted in various field including electronics, biotechnology, aerospace, automotive, life sciences, agricultural settings, and process industries [17]. The advantages offered by the RSM including reduced number of experimental trials, calculating the complex interaction between the independent variables, analysis, and optimization as well as the enhancement of existing design. Hence, optimization of an extraction process using RSM is important to examine suitable conditions to isolate bioactive compounds especially from different food matrices [18]. 

Thus, the present study was undertaken to optimize extraction conditions for higher total phenolic, flavonoids recovery, as well as determine in-vitro antioxidant activities of DPS extract using response surface methodology (RSM). Polyphenolic compounds from defatted seed extract of pitaya were identified by ultra-high-performance liquid chromatography-quadrupole time-of-flight mass spectrometry (UPLC-QTOF/MS). 

## 2. Results and Discussion

### 2.1. Fitting Model

Preference for the ideal extraction process which influenced better yield of total phenolic content (TPC), total flavonoid content (TFC), and antioxidant activity (ferric reducing antioxidant power (FRAP) and ABTS activity) of defatted pitaya seed extract (DPSE) was accomplished through response surface methodology. In the present study, the highest order polynomials were selected to choose the models where additional terms were significant and the models were not aliased in accordance to the sequential model sum of square. As suggested by the software, a quadratic polynomial model was chosen and well-fitted for all three independent parameters and responses [19]. The final predictive equations generated by the software were expressed in terms of coded factors as shown in Equations (1)–(4) where the empirical correlation between time of extraction (A) temperatures (B) and ethanol concentrations (C) were established:

Y(TPC) = + 109.53 + 2.66A + 17.60B + 9.23C − 13.83AB − 0.36AC − 3.96BC − 9.04A^2^ − 7.93B^2^ + 4.22C^2^ (1)

Y(TFC) = + 7.62 + 0.22A + 0.71B + 1.88C − 0.45AB − 0.97AC − 0.28BC − 0.24A^2^ + 0.11B^2^ − 0.18C^2^ (2)

Y(FRAP) = + 1.23 + 0.1152A + 0.2729B − 0.0245C + 0.0375AB − 0.1525AC − 0.0600BC − 0.0580A^2^ − 0.0050B^2^ − 0.0615C^2^ (3)

Y(ABTS) = + 91.85 + 3.82A + 3.69B + 0.8471C − 4.00AB − 2.37AC − 2.29BC − 2.25A² − 1.77B² + 0.2186C²(4)

The significance of the model was examined through analysis of variance (ANOVA) where a large F-value and small *p*-value of each term in the models implied a more significant impact on the respective response variables [20]. The ANOVA for second order polynomial model of TPC, TFC, and FRAP had shown that the model was significant (*p* < 0.05) with a *p*-value of < 0.0001 for TPC, TFC, FRAP, and ABTS activities (Table 1). Meanwhile, the coefficient of determination (R^2^) was employed to evaluate the quality of the fit quadratic model which were 0.9418, 0.9817, 0.9742, and 0.9840 for phenolic and flavonoid content, frap value, and ABTS activity respectively. This suggests that only 5.82%, 1.88%, 2.58%, and 1.60% of the total variations for TPC, TFC, FRAP, and ABTS activities respectively could not be explained by the model. The fitness of the model was identified through lack-of-fit test (*p* > 0.05), which indicated suitability of models to accurately predict the variations [21]. The coefficient variation (CV) is a measure of relative variability from the mean value, claimed that the model was reliable. In general, a CV of less than 10 percent revealed smaller variation in the mean value and indicated a better precision and reproducibility [22]. The CV values obtained for TPC, TFC, FRAP, and ABTS were 7.62, 4.62, 5.57, and 1.21 respectively, demonstrating the high reliability and accuracy of the model. 

### 2.2. Analysis of Response Surface

**Figure 1:**
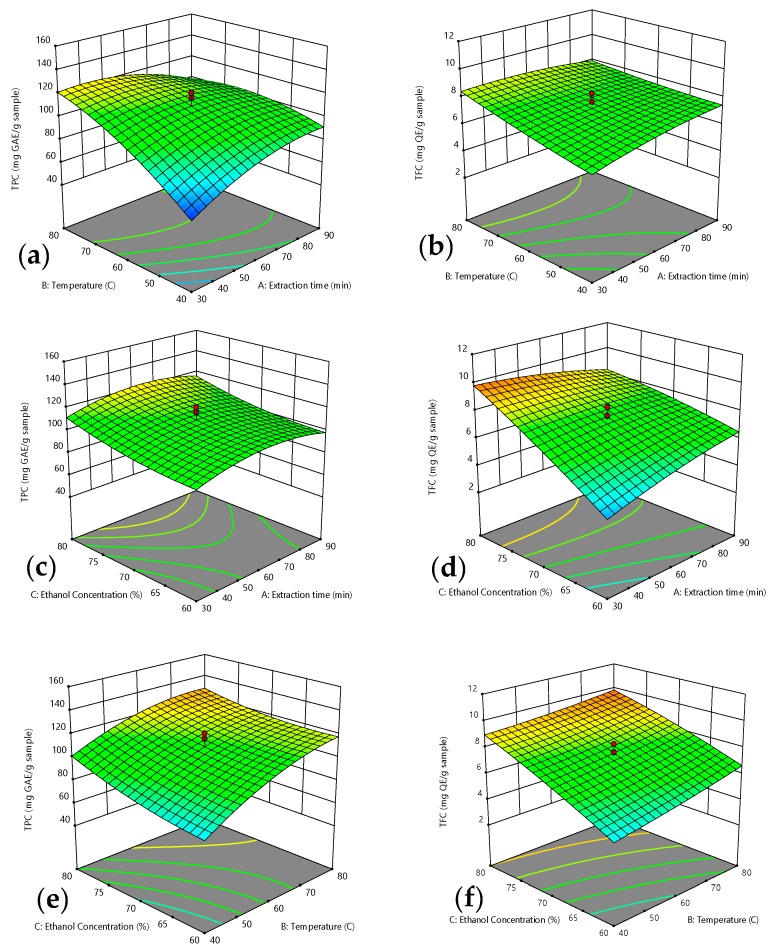
Response surface for: (**a**,**b**) effect of extraction time and temperature; (**c**,**d**) effect of extraction time and ethanol concentration, and (**e**,**f**) effect of temperature and ethanol concentration, on total phenolic content (TPC) (**a**,**c**,**e**) and TFC (**b**,**d**,**f**).

**Figure 2:**
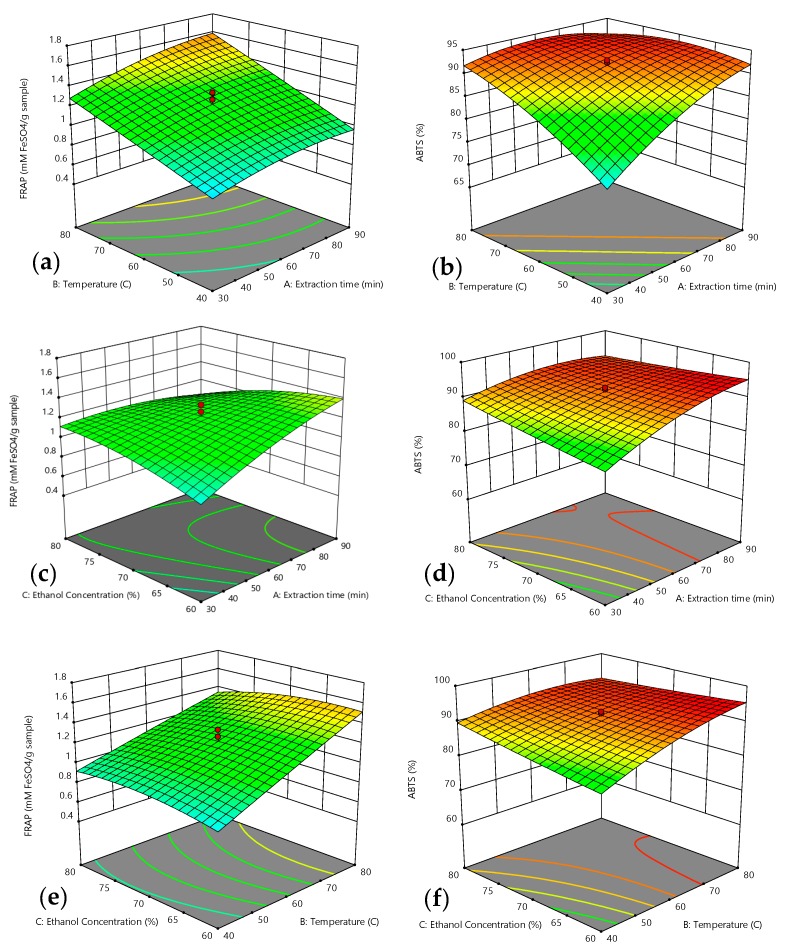
Response surface for: (**a**,**b**): effect of extraction time and temperature; (**c**,**d**): effect of extraction time and ethanol concentration; and (**e**,**f**): effect of temperature and ethanol concentration, on FRAP (**a**,**c**,**e**) and ABTS (**b**,**d**,**f**) activity.

Response surface methodology was used to estimate the recovery of TPC, TFC, FRAP, and ABTS activities based on varied values of tested factors, while the contours of the plots were used to establish their correlative interactions between the variables involved. According to [23], the best way to figure out the influence of the independent variables on the dependent variables are through drawing surface response plots of the model. The significance or not the mutual interaction between the factors can be determined by several shapes developed from the contour plots. A circular contour plot indicates the interactions between the corresponding parameters are negligible, while an elliptical contour or saddle nature implies the interactions between the corresponding parameters are significant [24]. The three-dimensional response surface and two-dimensional contours designed by the fitted model are demonstrated in Figure 1 and Figure 2. Each diagram shows the effect of two variables on yield of TPC, TFC, FRAP, and ABTS activities while holding other variables at their zero level.

### 2.3. Optimum Extraction Condition Based on TPC

The recovery yield of total phenolic content, TPC from defatted pitaya seed extract ranged from 52 to 144 mg GAE/g sample, which were higher than from kiwi fruit seeds [25] and defatted marigold *Tagetes erecta* L. residues [26]. In the present study, the mean recorded value was 100.83 mg GAE/g sample of the total extracts. The maximum yield of phenolic content was recorded for Experiment No. 18 whereas the lowest yield of flavonoid content discovered from Experiment No. 7. The ANOVA of the regression coefficient indicated that the two linear parameters, temperature (B) and ethanol concentration (C) were significant at (*p* < 0.0001 and *p* < 0.01) respectively. The quadratic (A^2^ and B^2^) and interactive effects between extraction time and temperatures (AB) were also significant (*p* < 0.05) on yield of total phenolic content. 

Figure 1a shows the interactions between extraction time and temperature on total phenolic contents at concentration of solvent fixed at 70%. It was observed that extraction efficiency was simultaneously enhanced with increase in extraction temperature and time. The recovery of phenolic contents increased rapidly with increase in extraction temperature during a shorter extraction time, while there was a slight increase in phenolic content with increase in extraction time at a higher extraction temperature. Under normal conditions, the temperature had constructive effect on extraction of phenolic compounds from plant sources [27,28,29]. The phenomenon could be clarified that higher temperature stimulates higher solubility of phenolic compounds in the extraction solvent. Similar trend on effect of higher temperature enhanced extraction efficiency of TPC was reported by [30,31] on an Indian medicinal plant and annatto seeds respectively. Higher temperature increases diffusion of extracted molecules, reduces its viscosity as well as improves mass transfer [32]. High temperatures from solvent have been reported to increase permeability of cell walls by breaking down interaction between phenolic compounds and macromolecules (proteins, polysaccharides) and thus facilitates recovery of phenolic yield in extract.

The interaction between extraction time and ethanol concentration (AC) revealed a significant (*p* < 0.05) positive effect on TPC as shown in Figure 1c. Yield of TPC gradually increased with increases in extraction time and ethanol concentration from 30–90 min and 60–80 °C respectively. However, prolonged extraction time did not significantly improve recovery of TPC. This occurrence could be explained by Fick’s second law of diffusion which stated that a final equilibrium is accomplished between the concentration of the solute in the solid matrix (plant matrix) and in bulk solution (solvent) after certain time [15]. Hence, a longer time does not necessarily extract more phenolic compounds. Extending extraction times might contribute to increase risk of phenolic oxidation unless reducing agents are added to the solvent system [33]. 

The effects of temperature and ethanol concentration at constant extraction time of 60 min increased recovery of TPC as shown in Figure 1e. The effect was probably due to the change in solvent polarity with the addition of certain amount of water to the solvent. Ethanol facilitated increase in TPC recovery by disrupting the bonding between the solutes and plant matrices, while water could enhance the swelling of cell material [34]. Previous findings reported that binary solvent system demonstrated higher yield of phenolic compounds and flavonoids as compared to mono-solvent system containing pure solvent or pure water [12]. Similar effects of this variables have also been reported for phenolic extraction from *Phaleria macrocarpa* (Scheff) Boerl fruits [22].

### 2.4. Optimum Extraction Condition Based on TFC

The recovery yield of total flavonoid content, TFC from defatted pitaya seed extract ranged from 2.69 to 10.67 mg QE/g sample, which were higher than grape byproducts [35]. The mean recorded value was 7.40 mg QE/g sample of total pitaya seeds extracts. Maximum yield of phenolic content was recorded from Experiment No. 15, whereas the lowest yield of flavonoid content was recovered from Experiment No. 7. The ANOVA of the regression coefficient indicated that all three linear parameters, (A, B, C), interaction parameters (AB, AC, BC) were significant at (*p* < 0.05). Meanwhile, only one quadratic effect A^2^ was significant at (*p* < 0.05) on yield of total flavonoid content. A 3D-surface plot of interaction between time (A) and temperature (B) at the fixed ethanol concentration of 70% is as shown in Figure 1b. The figure shows that TFC slightly increased with increase in extraction temperature. TFC increased until reaching an optimum temperature of 80 °C, similar with TPC. The effects of temperature on flavonoids extraction had previously been reported by various other researchers. For example, the highest flavonoid recovery was reported from *Flos populi* using solid-liquid extraction at temperature of 94.66 °C [36]. The achievement was supported by [30] and [37] who documented that elevated temperature enhanced better extraction yield. The effect was attributed to the fact that higher temperature disrupted the structure of plant matrix by weakening the phenolic matrix bonds which then increased the solubility of flavonoids [38]. Nevertheless, results of the present study demonstrated that yield of TFC decreased in long extraction time with increasing ethanol concentration as demonstrated in Figure 1d. Extraction time appeared to be one of the primary factors influencing an extraction process. It is therefore important in attempt at reducing energy cost in extraction procedure and in inhibiting the decomposition of active compounds. Extraction time can possibly be as short as few minutes or extended for up to 24 h [39]. This has been reported to be dependent on the extraction process phase, either rapid phase or slow phase [39]. Rapid phase is explained by the fact that solutes are present on surface sites of plant materials, and a slow phase corresponds to the molecular diffusion of the solute from internal sites through pores [29,40]. The present study achieved contrary to that recorded by [41] in extracting flavonoid from red and brown rice bran who observed an increase in flavonoid content with increase in time. These distinctions in time of extraction could be correlated to the nature of the sample (seed, leaf, rhizome, or bark), particle size, solvent type, and extraction approaches [29,42]. 

In the present study, the interaction effects between temperature (B) and ethanol concentration (C) show that TFC was found to be higher at higher values of variables as shown in Figure 1f. The maximum TFC recorded was when 80% ethanol concentration was used, compared to use of 70% ethanol with increasing extraction time. A general principle in solvent extraction is based on the law of similarity and intermiscibility “like dissolves like”, which means that solvents extract phytochemicals with a polarity value near to the polarity of the solvent [43]. The results suggest that the solvent polarity and the solubility of flavonoids compounds in pitaya seed extract were similar.

### 2.5. Optimum Extraction Condition on Antioxidant Activity

#### 2.5.1. Optimum Extraction Condition Based on FRAP Activity

The reducing power activity of DPSE ranged from 0.53 to 1.75 mM Fe^2+^/g sample. The mean recorded value was 1.14 mg mM Fe^2+^/g sample of total DPSE. The highest value was recorded from Experiment No. 20 whereas the lowest value was recorded from Experiment No. 7. The ANOVA on regression coefficient revealed that the two linear parameters, time (A) and temperature (B), interactive effect between time and ethanol concentration (AC) were significant at (*p* < 0.0001). Meanwhile, interaction parameter, (AB) and quadratic parameters (A^2^, B^2^) were significantly influenced at (*p* < 0.05). Similar effects of temperature have been previously recorded by other researchers on phenolic content of grape cane extracts which subsequently resulted in varied antioxidant activities [44]. The effects of mutual interaction between extraction parameters on the FRAP value of phenolic extract can be seen in Figure 2.

As shown in Figure 2a, the FRAP values increased as extraction time was prolonged up to 90 min. This patterns of responses were probably due to the fact that increasing extraction time provided longer contact of solids with the solvent and this enhanced the diffusion of phenolic compounds linked to the antioxidant activities [45]. Higher extraction time and medium ethanol concentration led to increase in FRAP values. However, extraction time was did not significantly influence total phenolic contents in defatted pitaya seed extracts which were in agreement with previous other reports by [46,47,48].

Figure 2c illustrates the interactive effect between extraction time and ethanol concentration. When ethanol concentrations were at 60%–80% and the extraction time at 30–90 min, the recorded value of FRAP increased initially and later decreased as the ethanol concentration was increased. This occurrence can be correlated to the changes in polarity of the compound that was responsible for reducing power activity. Therefore, the maximum value for reducing power could be obtained when the lowest ethanol composition was used. It has been reported that the polarity of the solvent used in extraction directly affects not only the quantity of total phenolic compounds, but also the composition and potency of the phenolic compounds as antioxidants [49,50,51]. Consequently, differences in extracted phenolic compounds result in varied antioxidant activity of the extracts.

The enhancement of FRAP activity significantly influenced by temperature. The FRAP value recorded in the present study gradually increased as extraction temperature was increased over time as shown in Figure 2e. The results implied that an increase in temperature led to an increase in antioxidant activity. This occurrence was probably due to the fact that at low temperatures, the rate of mass transfer was also low and additional time was needed for the phenolic compounds responsible for FRAP activity to dissolve in the solvent. Similar interactive effect showing similar response curve was also recorded by [52] on FRAP activity on tomatoes.

#### 2.5.2. Optimum Extraction Condition Based on ABTS Activity

The ABTS activity of the DPSE ranged from 69.79%–94.34%. The mean value recorded was 89.26% which was higher than ABTS activity reported for methanolic extract of *Adiantum caudatum* leaves [53]. The highest value was recorded for Experiment No. 20 whereas the lowest value was recorded from Experiment No. 1. The ANOVA of the regression coefficient revealed that the two linear parameters, time (A) and temperature (B), interactive effect between time and temperature (AB) and quadratic parameters (A^2^, B^2^) were significant at (*p* < 0.0001). Meanwhile, ethanol concentration (C), interaction parameter, (AC) and (BC) were significantly influenced at (*p* < 0.05). The effect between independent variables can be seen in Figure 2.

Figure 2b shows increases of ABTS activity of DPSE with increases in extraction time and temperature from 30–60 min and 40–60 °C respectively. Prolonged extraction time for up to 90 min and rise in temperature to 80 °C slightly decreased ABTS activity. The data revealed that the interactive effect between extraction time (A) and temperature (B) on ABTS activity was significant and in good agreement with the results shown in Table 1. Similar interaction was also recorded by [54] on ABTS activity of aqueous extract of *Schizophyllum commune*. Increase in ethanol concentration from 60–70% caused an increase in ABTS activity of DPSE (Figure 2d). This occurrence was probably due to the polarity of the solvent used coincided with the solubility of the phenolic compounds responsible for ABTS activity [22]. In the present study, the highest ABTS activity was recorded when DPS was extracted using 60% ethanol concentration. Similar trend was also obtained by [55], where 60% of ethanol concentration yielded the highest ABTS radical scavenging activity of *Dendropanax morbifera* (*D. morbifera*) Levillis leaves. Figure 2f shows the relationship between temperature and ethanol concentration on ABTS activity at a constant extraction time of 60 min. The data obtained revealed that ABTS activity was significantly influenced by increment in extraction temperature from 40 to 60 °C. This circumstance can be explained by the fact that solubility of solute and extraction efficiency were enhanced by increasing extraction temperature [56].

### 2.6. Optimization of Extracting Conditions for TPC, TFC, FRAP, and ABTS Activity

The numerical optimization for highest recovery of TPC, TFC, FRAP, and ABTS activity of DPSE were determined. The simultaneous optimization using desirability function approach proposed that the optimal extraction conditions for DPSE were at 45 min extraction time, 70 °C extraction temperature and 80% ethanol concentration at desirability of 93.8%. Therefore, extraction of DPS was performed based on the suggested extraction conditions and the data obtained were statistically compared with the predicted values given by the Design Expert 11.0 Software. Table 2 shows results from verification experiment which were in close agreement with predicted values at 93.8% confidence level.

### 2.7. Identification of Phytochemical Compound in DPSE

Separation of chemical constituents from DPSE in negative ionization mode was analyzed by ultra-high-performance liquid chromatography coupled with quadrupole time-of-flight mass spectrometry (UPLC-QTOF/MS) method. A summary of identified potential compounds are shown in Table 3. The identification of the detected compounds was made by comparing retention times MS data (neutral and observed mass) and theoretical fragmentation with data reported in literature. The isolated compounds were then categorized as tentative or confirmed. For the tentative category, they were assigned to a compound identified in Waters library with acquisition mass accuracy less than 5 ppm with at least one fragment ion [57]. The confirmed category assigned to a compound identified from Water Library and compared with available standard sample of the same compound. Based on the requirement set for UPLC-QTOF/MS method, seven different compounds were detected with 6 of them tentatively categorized and only rutin was categorized as confirmed and validated with the standard. Within these analyzed compounds, five were classified under flavonoid group while two were classified as phenolic acid group.

**Figure 3:**
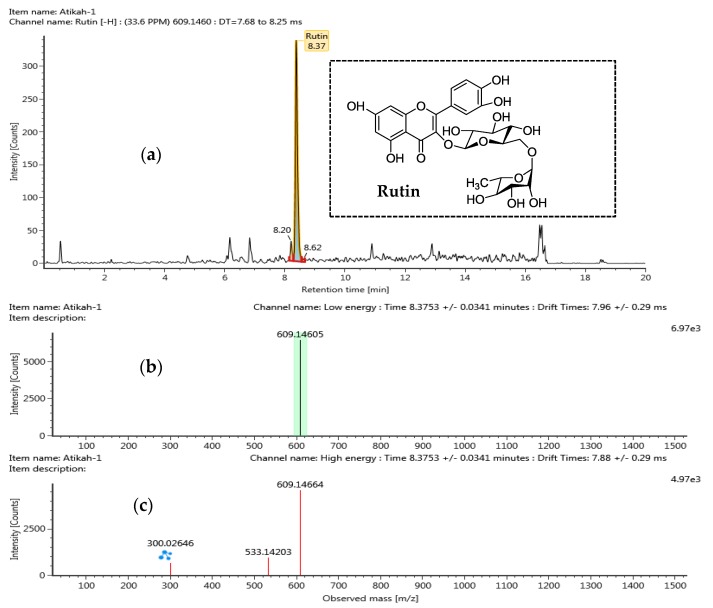
UPLC-QTOF/MS chromatograms of Rutin from defatted pitaya seed extract (DPSE): (**a**) mass spectra (MS/MS) of Rutin obtained at low collision energy; (**b**) mass spectra (MS/MS) of Rutin obtained at high collision energy (**c**).

Rutin is a common flavonoid found in seed extracts such as in the *Euryale ferox* seed shells [58], baobab seeds extract, grape seeds [59], and buckwheat seeds [60]. It was previously reported to possess health benefits and considered as being a good antioxidant agent [58]. MS/MS Spectra of Rutin obtained at low and high collision energy is shown in Figure 3. Different in collision energy is a technique used by mass spectrometry to induce fragmentation of selected ions in the gas phase which can then be analyzed by mass spectrometry [61]. Low collision energy is referring to collisions where the precursor ions have kinetic energies less than 1 kiloelectron volt (1 keV) or in the range of a few eV to a hundred eV. High collision energy referring to the collision where the precursor ion is accelerated to kinetic energies in the kilovolt range normally from 1 keV to 20 keV. Low collision energy led to rearrangement of the ion structure, meanwhile, high collision energy can produce more fragmentation that are not formed in low collision energy. Hence, more structural information can be obtained and easier for result interpretation [62].

For other flavonoids 2, 3, 4, and 5 occurred as O-glycoside with sugar bound at certain position. The identified flavonoids in DPSE included the following:

- kaempferol-3-O-rutinoside,
- kaempferol-3-O-β-d-glucopyranoside,
- apigenin-7-O-α-l-rhamnose(1->4)-6″-O-acetyl-β-d glucoside, and
- isohamnetin-3-O-(2G-α-l-rhamnosyl)-rutinoside.

Most of the flavonoids contained either kaempferol, apigenin or isohamnetin core moieties. These illustrate that sugar moieties mostly attached to the basic skeleton flavonoid of DPSE and the total number of sugar moieties and their attachment to the structure of flavonoid effect their antioxidant activity [63]. In addition to the above, phenolic acid metabolites such as sinapic acid and E-p-coumatic acid have been detected in DPSE. These metabolites have been reported found in extract of plant family of *Cactaceae* and reported to have anti-inflammatory activities [64].

## 3. Materials and Methods

### 3.1. Materials

Sodium carbonate (Na_2_CO_3_), Folin–Ciocaltue’s reagent, gallic acid, quercetin, aluminium chloride (AlCl_3_), iron (II) sulfate 7-hydrate (FeSO_4_·7H_2_O), iron (III) chloride 6-hydrate (FeCl_3_·6H_2_O), acetic acid, 2,2,6-Tri(2-pyridyl)-*s*-triazine (TPTZ), and hydrochloric acid (HCl) were purchased from Sigma-Aldrich. Only analytical grade of chemicals was used throughout the experiments. Fruits of *Hylocereus polyrhizus* were obtained from a local farm located in Sepang, Malaysia. Seeds were separated from the pulp and washed under running tap water to remove the mucilage and dried in the laboratory. Dried seeds were ground into smaller particles and kept in air-tight bottles. Subsequently, the seeds were defatted by maceration method using *n*-hexane as the solvent. The defatted seeds were left overnight in a fume hood to let residual *n*-hexane evaporates before being used in subsequent polyphenols extraction procedures.

### 3.2. Extraction of Defatted Pitaya Seed (DPS)

An amount of 3 g of defatted seeds was mixed with extracting solvent at different levels of independent variables including ethanol concentration (60%–80% *v*/*v*), extraction time (30–90 min) and extraction temperature (40–80 °C) in separate conical flasks and placed in a thermostatic water bath set at a constant shaking of 90 rpm. Parafilm and aluminum foil were used to cover the conical flasks to prevent loss of solvent due to evaporation during the extraction process. The mixture was subsequently centrifuged at 10,000 rpm for 5 min to separate the insoluble materials. The supernatant was filtered using Whatman No. 1 filter papers and vacuum-dried in a rotary evaporator at 60 °C until the solvent was completely removed. All the samples were stored at −4 °C until further analyses.

### 3.3. Experimental Design

A five-level with three independent variables central composite design (CCD) was employed to determine the optimal extraction condition of defatted pitaya seed (DPS). The experiment was designed using Design-Expert software version 11.1.0.1 (Stat-Ease, MN, USA). Three independent variables (extraction times, temperatures and ethanol concentrations) were selected and their coded values and levels of each variable are presented in Table 4. A total of 20 experimental runs were conducted to determine TPC, TFC and in vitro antioxidant activity based on FRAP and ABTS activity (Table 5). Least square regression was used by CCD to fit the experimental data to a second-degree polynomial model. The model was explained by the following equation:

$$Y=A_{0}+\;\overset{k}{\underset{i=1}{\sum }}A_{i}X_{i}+\overset{k}{\underset{i=1}{\sum }}A_{ii}X_{i}^{2}+\overset{k-1}{\underset{i=1}{\sum }}\overset{k}{\underset{j=i+1}{\sum }}A_{ij}X_{i}X_{j}$$

where *Y* is the predicted response, *A_0_*, *A_i_*, *A_ii_*, *A_ij_* are the constant, linear coefficient, quadratic coefficient, and interaction coefficient respectively. *X_i_* and *X_j_* are independent variables. *k* is the number of variables. 

### 3.4. Determination of Total Phenolics Content (TPC)

Total phenolic content was determined using method described by [65] with several modifications. Firstly, an amount of 100 µL (1 mg/mL) of sample extract was transferred into test tubes. Then, 50 µL of Folin solution previously diluted with 7.9 mL distilled water was added. After 4 min, 1.5 mL of 7.5 *w*/*v*% sodium carbonate solution was added to the sample tubes and incubated for 2 h in a dark room at room temperature. The absorbance values of the samples were recorded at 765 nm using a UV-VIS microplate reader. All the analyses were performed in triplicates to obtain the main value of the absorbance. Gallic acid at different concentrations was prepared to establish a standard calibration curve. TPCs in the sample extract were calculated by referring to the standard calibration curve and expressed as mg gallic acid equivalent (mg GAE/g) of extract sample. 

### 3.5. Determination of Total Flavonoid Content (TFC)

Flavonoids content in the sample extract was assayed using spectrophotometric method as described by [66] with slight modifications. An amount of 100 µL (1 mg/mL) of sample and 100 µL of 2% AlCl_3_ were mixed and incubated at room temperature for 15 min. Increase in absorbance was determined using UV-Vis microplate reader at λmax = 415 nm. The same procedure was repeated for the standard solution of quercetin to obtain the standard calibration curve. TFC was calculated based on quercetin calibration curve and expressed as mg quercetin equivalent (mg QE/g) of extract.

### 3.6. Ferric Reducing Antioxidant Power (FRAP)

FRAP assay was conducted following method of [22] with minor modifications. Firstly, acetate buffer (330 mM, pH 3.6), a 10 mM TPTZ solution in 40 mM of HCl and 20 mM FeCl_3_·6H_2_O solution were mixed in ratio of 10:1:1 (*v*/*v*) to prepare for FRAP reagent. The working FRAP reagent was freshly prepared prior to use. An amount of 60 µl of the sample extract was mixed with 1.8 ml FRAP reagent and the increase in absorbance was measured in comparison to a blank at 593 nm after 4 min. A standard curve was constructed using Fe_2_SO_4_ solution ranging from (0.1–1.0 mM) and the results were expressed as mM Fe2+/g dry weight.

### 3.7. ABTS Radical Scavenging Activity

The ABTS radical scavenging activity of the sample was carried out according to the method [67] with minor modification. Firstly, ABTS radical cation was prepared by reacting 7 mM ABTS with 2.45 mM potassium persulfate in ethanol and kept the mixture in the dark room temperature for 12–16 h before use. Then, the solution was diluted with ethanol to obtain an absorbance of 0.70 (±0.02) at 734 nm. For the evaluation of antioxidant activity, 2 mL of ABTS radical solution were mixed with 20 µL of the sample. The absorbance changes at 734 nm were measured using UV-Vis microplate reader after 30 min of the initial mixing. Antioxidant activity as the percent inhibition of absorbance at 734 nm was calculated using the following equation:

ABTS·+ scavenging activity = % inhibition: ((AB − AA))/AB) × 100(5)

where, AB is the absorbance of ABTS radical with ethanol; AA is absorbance of ABTS radical + sample.

### 3.8. Identification of Phytochemical Compounds in Defatted Pitaya Seed Extract (DPSE)

Phytochemical compounds in DPSE were identified using ultra-high-performance liquid chromatography (UPLC-MS). The analysis was completed with Waters Acquity ultra-performance LC system (Waters, Milford, MA, USA). Chromatographic separation was done using a column (ACQUITY UPLC HSS T3, 100 mm × 2.1 mm × 1.8 µm, Waters, Manchester, UK. The UPLC systems was connected to Vion IMS QTof detector (Waters, Milford, MA, USA). The mobile phase used were 0.1% formic acid (A) and acetonitrile (B). The mobile phase composition consisted of the following multistep linear gradient: 0 min, 1% B and 99% A; 0.5 min, 1% B and 99% A; 16.00 min, 35% B and 65% A; 18.00 min, 100% B and 0% A; and 20.00 min, 1% B and 99% A. The injection volume of the sample was 1 µL. The flow rate was set at 0.6 mL/min. The data were obtained in the range of *m*/*z* 50–1500 at 0.1 s/scan in high-definition mass spectrometry elevated energy (HDMSE) with collision energies (CE) at a fixed 4 eV and at ramped from 10 to 40 eV were required for low energy and high energy scan respectively. 

## 4. Conclusions

Central composite design (CCD) response surface methodology was employed to evaluate the optimized extraction process for the recovery of TPC, TFC. Antioxidant activity was based on FRAP and ABTS from DPSE by analyzing the interaction effects between the independent variables (extraction time, extraction temperature and ethanol concentration). The results revealed that, extraction temperature significantly influenced the extraction process of DPS. The extraction time at 45 min with extraction temperature at 70 °C and 80% of solvent concentration which resulted in 128.58 ± 1.61 mg GAE/g sample, 9.805 ± 0.69 mg QE/g sample, 1.23 ± 0.03 mM Fe^2+^/g sample, and 91.62% ± 0.15 were found to be the optimized condition for experimental run. Results from the validation experiments were in good agreement with the predicted values. Results from UPLC-QTOF/MS revealed that there were seven phytochemicals identified in DPSE with flavonoid found to be the major compound. The optimized extraction method was helpful in designing the experiment, separation of the chemical compounds which then contributed to further work. 

## Figures and Tables

**Table 1:** Analysis of Variance (ANOVA) of regression equation for optimization of TPC, TFC, FRAP, and ABTS.

Source	TPC				TFC				FRAP				ABTS			
	dF^a^	SS^b^	F-Value	*p*-Value	dF^a^	SS^b^	F-Value	*p*-Value	dF^a^	SS^b^	F-Value	*p*-Value	dF^a^	SS^b^	F-Value	*p*-Value
Model	9	9533.98	17.97	<0.0001	9	67.3	63.87	<0.0001	9	1.53	41.97	<0.0001	9	722.71	68.43	<0.0001
A	1	96.90	1.64	0.2288	1	0.6602	5.64	0.039	1	0.1811	44.82	<0.0001	1	199.29	169.83	<0.0001
B	1	4231.34	71.76	<0.0001	1	6.95	59.39	<0.0001	1	1.02	251.6	<0.0001	1	185.83	158.36	<0.0001
C	1	1163.51	19.73	0.0013	1	48.33	412.77	<0.0001	1	0.0082	2.03	01849	1	9.8	8.35	0.0161
AB	1	1530.98	25.96	0.0005	1	1.66	14.15	0.0037	1	0.0112	2.78	0.1262	1	128.24	109.28	<0.0001
AC	1	1.00	0.0170	0.8989	1	7.57	64.63	<0.0001	1	0.1861	46.04	<0.0001	1	44.79	38.17	0.0001
BC	1	125.37	2.13	0.1755	1	0.6161	5.26	0.0447	1	0.0288	7.13	0.0235	1	42	35.79	0.0001
A^2^	1	1178.75	19.99	0.0012	1	0.8306	7.09	0.0238	1	0.0485	11.99	0.0061	1	72.79	62.03	<0.0001
B^2^	1	906.72	15.38	0.0029	1	0.1742	1.49	0.2505	1	0.0004	0.0874	0.7736	1	45.34	38.64	<0.0001
C^2^	1	256.80	4.36	0.0635	1	0.4853	4.15	0.0691	1	0.0545	13.5	0.0043	1	0.6885	0.5867	0.4614
Residual	10	589.64			10	1.17			10	0.0404			10	11.73		
Lack of fit	5	323.64	1.22	0.4174	5	0.4582	0.6431	0.6801	5	0.0129	0.4677	0.788	5	9.07	3.41	0.1022
R^2^		0.9418				0.9829				0.9742				0.9840		
R^2^_Adj_		0.8893				0.9675				0.9510				0.9696		
CV		7.62				4.62				5.57				1.21		

dF^a^: degree of freedom; SS^b^: sum of square, TPC: total phenolic content; TFC: total flavonoid content; FRAP: ferric reducing antioxidant power (antioxidant activity); ABTS: 2,2′-azino-bis (3-ethylbenzothizoline-6-sulfonic acid (anti-oxidant activity).

**Table 2:** Predicted and Experimental Values for Responses of TPC, TFC, FRAP, and ABTS.

	TPC (mg GAE/g Sample)	TFC (mg QE/g Sample)	FRAP (mM Fe^2+^/g Sample)	ABTS (%)
Predicted	129.75	9.995	1.24	92.87
Experimental	128.58 ± 1.61	9.805 ± 0.69	1.23 ± 0.03	91.62 ± 0.15

TPC: total phenolic content; TFC: total flavonoid content; FRAP: ferric reducing antioxidant power (antioxidant activity); ABTS: 2,2′-azino-bis(3-ethylbenzothizoline-6-sulfonic acid (antioxidant activity).

**Table 3:** Compounds identified in defatted Pitaya, *Hylocereus polyrhizus* seed extract using UPLC-QTOF/MS.

No.	A	Component Name	B	C	D	Identification Status and Category
1	FL	Rutin	610.15338	609.146	8.38	Identified, confirmed
2	FL	Kaempferol-3-O-rutinoside	594.15847	593.1514	6.67	Identified, tentative
3	FL	Kaempferol-3-O-β-d-glucopyranoside	448.10056	447.093	9.32	Identified, tentative
4	FL	Apigenin-7-O-α-l-rhamnose (1→4)-6”-O-acetyl-β-d-glucoside	620.17412	619.1664	7.98	Identified, tentative
5	FL	Isorhamnetin-3-O-(2G-α-l-rhamnosyl)-rutinoside	770.22694	769.2208	7.74	Identified, tentative
6	PA	Sinapic acid	224.06847	223.0609	5.42	Identified, tentative
7	PA	E-p-Coumatic acid	164.04734	163.0396	11.69	Identified, tentative

A: compound class; FL: flavonoid; PA: phenolic acid, B: natural mass (Da); C: observed *m*/*x*; D: retention time (Min.). UPLC-QTOF/MS: ultra-high-performance liquid chromatography coupled with quadrupole time-of-flight mass spectrometry.

**Table 4:** Independent variables and their levels in central composite design.

Independent Variables	Levels				
	−α	−1	0	1	−α
Extraction time (A) (min)	−9.54	30	60	90	110.45
Temperature (B) (°C)	26.36	40	60	80	93.64
Ethanol concentration (C) (%)	53.18	60	70	80	86.82

**Table 5:** Experimental design with observed responses of total phenolic content (TPC), total flavonoid content (TFC), and ferric reducing antioxidant power (FRAP) from defatted pitaya seed extract.

Run	Extraction Time, A	Temperature, B	Ethanol Conc., C	TPC	TFC	FRAP	ABTS
	(Min.)	(°C)	(%)	(mg GAE/g)	(mg QE/g)	(mM Fe^2+^/g)	(%)
1	9.54622	60	70	75.83	6.38	0.85	78.89
2	60	60	70	103.75	7.34	1.34	91.22
3	60	26.3641	70	55.83	6.65	0.77	80.87
4	90	80	80	107	8.54	1.34	89.13
5	110.454	60	70	89.63	7.44	1.25	91.9
6	30	80	60	127.75	5.96	1.21	91.97
7	30	40	60	52	2.69	0.53	69.79
8	90	40	80	102.42	8.2	0.75	92.13
9	60	60	70	105.75	7.64	1.12	91.08
10	60	60	70	102.75	8.29	1.22	92.35
11	60	60	53.1821	96.5	3.88	1.08	91.52
12	60	60	70	108.38	7.2	1.19	91.35
13	60	93.6359	70	115.92	9.15	1.63	92.6
14	60	60	70	116.25	7.68	1.22	92.33
15	30	80	80	126.75	10.67	1.28	92.37
16	90	40	60	89	6.27	1.05	92.03
17	60	60	70	120.75	7.55	1.27	92.82
18	60	60	86.8179	144	10.26	1	93.22
19	30	40	80	77.5	9.35	0.97	83.21
20	90	80	60	98.75	6.88	1.75	-
